# Combating iron and zinc malnutrition through mineral biofortification in maize through plant growth promoting *Bacillus* and *Paenibacillus* species

**DOI:** 10.3389/fpls.2022.1094551

**Published:** 2023-02-01

**Authors:** Maqshoof Ahmad, Azhar Hussain, Abubakar Dar, Muhammad Luqman, Allah Ditta, Zafar Iqbal, Hafiz Tanvir Ahmad, Farheen Nazli, Walid Soufan, Khalid Almutairi, Ayman El Sabagh

**Affiliations:** ^1^ Department of Soil Science, The Islamia University of Bahawalpur, Bahawalpur, Pakistan; ^2^ Department of environmental science, Shaheed Benazir Bhutto University, Sheringal, Pakistan; ^3^ School of Biological Sciences, The University of Western Australia, Perth, WA, Australia; ^4^ National Research Center of Intercropping, The Islamia University of Bahawalpur, Bahawalpur, Pakistan; ^5^ Provincial Reference Fertilizer Testing Laboratory, Raiwind Lahore, Lahore, Pakistan; ^6^ Institute of Agroindustry and Environment, The Islamia University of Bahawalpur, Bahawalpur, Pakistan; ^7^ Plant Production Department, College of Food and Agriculture Sciences, King Saud University, Riyadh, Saudi Arabia; ^8^ Department of Agronomy, Faculty of Agriculture, University of Kafrelsheikh, Kafr el-Sheikh, Egypt

**Keywords:** food security, undernourished, vitamins, biofortification, PGPR

## Abstract

**Introduction:**

The burgeoning population of the world is causing food insecurity not only by less food availability but also by the malnutrition of essential nutrients and vitamins. Malnutrition is mostly linked with food having micronutrients lower than the optimal concentration of that specific food commodity and becoming an emerging challenge over the globe. Microbial biofortification in agriculture ensures nutritional security through microbial nitrogen fixation, and improved phosphate and zinc solubilization, which increase the uptake of these nutrients. The present study evaluates the novel plant growth-promoting rhizobacteria (PGPR) to biofortify maize gain.

**Methods:**

For this purpose, a pot and two field experiments for maize were conducted. PGPRs were applied alone and in combination for a better understanding of the biofortification potential of these strains. At physiological maturity, the growth parameters, and at harvest, the yield, microbial population, and nutritional status of maize were determined.

**Results and discussion:**

Results revealed that the consortium (ZM27+ZM63+S10) has caused the maximum increase in growth under pot studies like plant height (31%), shoot fresh weight (28%), shoot dry weight (27%), root fresh (33%) and dry weights (29%), and microbial count (21%) in the maize rhizosphere. The mineral analysis of the pot trial also revealed that consortium of ZM27+ZM63+S10 has caused 28, 16, 20, 11 and 11% increases in P, N, K, Fe, and Zn contents in maize, respectively, as compared to un-inoculated treatment in pot studies. A similar trend of results was also observed in both field trials as the consortium of ZM27+ZM63+S10 caused the maximum increase in not only growth and biological properties but also caused maximum biofortification of mineral nutrients in maize grains. The grain yield and 1000-grain weight were also found significantly higher 17 and 12%, respectively, under consortium application as compared to control. So, it can be concluded from these significant results obtained from the PGPR consortium application that microbial inoculants play a significant role in enhancing the growth, yield, and quality of the maize. However, the extensive evaluation of the consortium may help in the formulation of a biofertilizer for sustainable production and biofortification of maize to cope with nutritional security.

## 1 Introduction

Cereals are the most planted crops and common person’s food all over the world. Maize provides vitamins, starch, fiber, protein, and sugar; 100 g of maize grains contains 361 calories. Maize at its vegetative stage is also used as animal feed/fodder and provides the chief source of energy for livestock and poultry feeding (Arain, 2013). Due to the rapid increase in the world’s population, the food demand has increased and hence cereals production needs to be increased with every passing day. However, the annual yield and productivity of cereals are declining in the developing world mainly in Asia. The use of balanced inputs (fertilizers, pesticides, herbicides) in the recent era is the key to gaining maximum production of cereals (Pingali and Heisey, 2001). The reason behind the lower production of maize in Pakistan includes water scarcity, non-availability at critical stages and the rising cost of chemical (fertilizers and pesticides), and the availability of certified hybrid seed in the market. Moreover, climate change is causing a 15-20% decline in the production of hybrid varieties to their actual potential (Ayub et al., 2021). Furthermore, climate change is posing serious threats to crop production in developing countries like Pakistan and shifting them from food surplus to food dearth countries (Abbas, 2022).

Imbalanced utilization of macronutrients, diminished use of organic fertilizers and natural manure, decreased incorporation of plant residues, and exhaustive maize yield in the previous decade have caused micronutrient inadequacies in the soils of Pakistan (Bashir et al., 2021). The greater parts of the soil in maize growing territories of Pakistan have turned out to be inadequate in Zn and Fe. Microorganisms in combination with chemical and organic fertilizers are increasing the uptake of micronutrients, which results in a higher concentration of micronutrients particularly Fe and Zn in plants (Steven, 1991; Mishra et al., 2004; Shivay et al., 2010). The nitrogen requirements of maize are increasing to get higher yields of maize. Balanced nitrogen application along with other macro/micronutrients is important for acquiring efficient grain yield and quality (Zebrath et al., 2009). Nitrogen deficiency is the most significant yield-limiting factor for grain crops (Shah et al., 2003). The use of phosphorus alongside *Rhizobium* inoculation improves root and plant development and ultimately yield of crops (Gentili and Huss-Danell, 2003; Fatima et al., 2007). Qualities of oat, for example, grain yield, chlorophyll, and protein contents were improved when PGPR were associated with roots (Tawfiq and Ahmad, 2014). Bio-fertilizers are helpful in the incorporation of microbes in the soil that colonize plant roots and expedite plant development by various mechanisms (Glick, 1995). The utilization of PGPR is consistently expanding in agribusiness because it enhances fertilizer use efficiency, decreases fertilizer requirements for crops, and in addition utilization of synthetic pesticides and different agrochemicals (Rana et al., 2012). The PGPR can enhance the uptake and micronutrient biofortification (N, P, Fe, Zn, and Cu) in cereal grains, through nutrient solubilization, siderophores, and exopolysaccharides production (Jalal et al., 2021; Shahane and Shivay, 2022). Biofortification through rhizobacteria has gained popularity to improve Zn and other micronutrient contents in grain crops. For instance, Gopalakrishnan et al. (2016) revealed the species from the genera *Pseudomonas, Brevibacterium, Bacillus, Enterobacter, and Acinetobacter* as potential candidates for biofortification and biocontrol in plants. These microorganisms also can incorporate micronutrients inside eatable plant tissues through solubilization of their indigenous insoluble sources present in the soil (Khalid et al., 2015; Gopalakrishnan et al., 2016). The Consultative Group on International Agricultural Research (CGIAR) has reported the hereditarian potential for increment in bioavailable Fe and Zn contents in grain crops for example; rice, wheat, and maize after harvest (Cakmak et al., 2010; de Santiago et al., 2011; Galindo et al., 2021).

The utilization of PGPR for biofortification not just encourages us to manage the issue of malnutrition among the population, in addition, improves cereals yield, soil fertility, and biodiversity (Bouis et al., 2003; White and Broadley, 2005; Ditta et al., 2022; Jalal et al., 2022). The soil microbial community is a solid pole to assess soil richness and directs the accessibility of supplements to harvest plants. Any change in the microbial community in this manner can influence the supplement take-up by harvest plants (Nuttall et al., 2017). The presence of Zn in the soil is not an issue in soils but its availability to plants remains an issue throughout the growing season of crops and hence lowers their fortification and fertility status of the soil (Sharma et al., 2013; Majeed et al., 2022). However, the Zn deficiency in Pakistani soils is mainly due to the calcareous and basic nature of our soils. The applied Zn solubilizing PGPR populations help in this regard to solubilize Zn in plant-available form and its uptake by plants (Rana et al., 2012). These microbes help plants to take up more Zn from the soil through Zn solubilization through organic acids secretions (Hussain et al., 2015) and expanding the surface area of roots through the production of auxins in the rhizosphere. Maize is the staple diet of most of the population of the world including Pakistan. Keeping in view the discussion above, it can be hypothesized that the sole and combined application of PGPR may have the potential to improve maize yield and quality. The objective of the present investigation is to biofortify maize by pre-isolated and characterized Zn solubilizing PGPR strains without any harmful effects on the environment from chemical fertilizer application and to cope with nutritional insecurity.

## 2 Materials and methods

### 2.1 Collection of bacterial strains

Pre-isolated Zn solubilizing and siderophore-producing bacterial strains *Bacillus subtilis*. ZM63, *Bacillus aryabhattai*. ZM31, *Bacillus aryabhattai*. S10 and *Paenibacillus polymyxa*. ZM27, having accession numbers KX788861, KX788860, KX788862, and KX788859, respectively, (Najm-ul-Sehar et al., 2015; Mumtaz et al., 2017) were taken from the Soil Microbiology and Biotechnology Laboratory, Department of Soil Science, The Islamia University of Bahawalpur. The strains were tested in the present study to investigate their potential for Fe and Zn biofortification in maize.

### 2.2 Preparation of consortium

Fresh cultures of each bacterial strain were inoculated in Luria-Bertani broth medium prepared in a 250 mL conical flask and kept in a shaking incubator at 30 ± 1°C for 48 h. After incubation, equal volumes from each strain (having optical density OD600 = 0.65) were mixed and vortexed for 1 min for homogenization of the inoculum to make a consortium as described by Dar et al. (2020).

### 2.3 Seed disinfection and inoculation

Hybrid seeds of the maize were soaked in sodium hypochlorite solution (5%) for 2 min followed by 30 s dipping in the 70% ethanol and rinsing six times with distilled autoclaved water for removal of chemicals from the seed surface. The surface sterilized seeds of the maize were moistened with 10% sugar solution and coated by slurring in a 4:5 carrier-to-inoculum ratio for single as well as co-inoculation, however, the control treatment was prepared by using sterilized Luria-Bertani broth (Zahir et al., 2018).

### 2.4 Pot trial

A pot experiment was carried out in the wirehouse of the Soil Science Department, at the Islamia University of Bahawalpur. The atmosphere of Bahawalpur is dry with normal precipitation is under 250 mm and the soil is dominated by Aridisols order as per the taxonomical classification of USDA (Soil Survey Staff, 2006). Soil from the field was collected, air-dried, sieved, and filled in the pots at 12 kg soil per pot. Before planting, the soil sample was taken, dried, blended, sieved, and examined for the physicochemical attributes of the soil (**Table S1**). The treatments (T_0_=Control, T_1_=ZM27, T_2_= ZM31, T_3_=ZM63, T_4_=S10, T_5_=ZM27+ZM31, T_6_=ZM27+ZM63, T_7_=ZM27+S10, T_8_=ZM31+ZM63, T_9_=ZM31+S10, T_10_=ZM63+S10, T_11_=ZM27+ZM31+ZM63, T_12_=ZM27+ZM31+S10, T_13_=ZM27+ZM63+S10, T_14_=ZM31+ZM63+S10, T_15_=ZM27+ZM31+ZM63+S10) in pots were arranged in a completely randomized design (CRD) replicated thrice. Full-recommended doses of P and K (90 kg ha^-1^ and 60 kg ha^-1^, respectively) and one-third of the N (120 kg ha^-1^) were applied before sowing as di-ammonium phosphate (46% P_2_O_5_ and 18% N), sulfate of potash (50% K_2_O), and urea (46% N), respectively. Whereas the remaining nitrogen was applied in two splits at the tillering and physiological maturity/flowering. After 70 days, the crop was reaped for growth parameters. Shoot samples were gathered, air-dried, ground, and stored for mineral determination.

### 2.5 Field trials

The validity of the pot experiment results was tested through field experimentation. Two field experiments were carried out in both seasons at the research farm, Department of Soil Science, the Islamia University of Bahawalpur. The treatments were arranged in a randomized complete block design (RCBD), with three blocks assumed as replications with an experimental unit size of 5x5 ft^2^. These field trials were conducted with similar treatments as those used in the pot experiment. The fertilizer requirements and application were also done as in pot experimentation. The maize crop was irrigated by available canal water. After maturity crop was harvested for growth and yield parameters. Growth parameters were noted at the time of harvesting, after that plant and grain samples were prepared to analyze for N, P, and K.

### 2.6 Nutrient analysis in shoots and grains

The plant samples were digested as per Wolf’s method (Wolf, 1982). Oven-dried and ground plant shoot and grain samples (0.1 g) were taken in a conical flask and placed overnight after adding 5 mL of concentrated H_2_SO_4_ at room temperature in a fume cabinet. Overnight incubated samples were spiked with 1 mL H_2_O_2_ (35%) before heating on a hot plate at 350 °C. The process of addition of H_2_O_2_ and heating was repeated until colorless/milky appearance. The material was filtered and diluted up to 50 mL with distilled water. The filtrate was used for the determination of Nitrogen through the Kjeldahl apparatus, phosphorus on the spectrophotometer, potassium on the flame photometer, and iron and zinc on the atomic absorption spectrophotometer following Ryan et al. (2001).

### 2.7 Bacterial population (CFU × 10^4^) in the rhizosphere

The rhizospheric microbial population was determined from the rhizosphere soil samples taken at harvesting. These samples were immediately shifted to the laboratory and placed at 4°C until analyzed. These samples were analyzed for bacterial population (cfu/g soil) through serial dilution and spread plate technique using general purpose medium (GPM). The inoculated plates were placed in an incubator at 30 ± 2°C for 48h. Afterward, the colonies from the dilution ranging between 30-300 were counted and expressed in scientific notation as per the method described by Alexander (1982).

### 2.8 Statistical analysis

Data obtained from pot and field trials was computed statistically for significance through one-way ANOVA, respectively, on Statistix 8.1^®^ computer-based software (Steel et al., 1997). However, the difference among treatment means was computed by applying Least Significance Difference (LSD) test at 5% probability (Montogomery, 2013).

## 3 Results

To evaluate the effectiveness of a novel bioinoculant for the biofortification of maize, a pot experiment and two field experiments were conducted at a wirehouse and research area of the Department of Soil Science, the Islamia University of Bahawalpur, respectively. The pre-identified and compatible PGPR strains ZM27 (KX788859), ZM31 (KX788860), ZM63 (KX788861), and S10 (KX788862) and their possible combination were tested in pot and field trials to determine the role of these bacterial strains in zinc and iron uptake and biofortification in maize grin to fulfill the nutritional requirement of zinc of the burgeoning population through natural sources.

### 3.1 Pot trial

#### 3.1.1 Growth promotion of maize by PGPR application

Plant height and shoot fresh biomass were significantly enhanced because of the inoculation/co-inoculation of PGPR strains as compared to the un-inoculated control (**Table 1**). Single as well as consortium application of the bacterial strains had an impact on plant height and shoot fresh biomass of maize in a pot experiment. Plant height was significantly enhanced under sole inoculation of PGPR strains except for ZM27, which has caused a non-significant increase in plant height, to the un-inoculated control. Maximum plant height was caused in treatment receiving PGPR consortium (ZM27+ZM63+S10) followed by co-inoculation (ZM27+S10) causing 31 and 28%, increases as compared to control, respectively. Similar findings were also observed under shoot fresh biomass where ZM27+ZM63+S10 has caused a 28% increase in shoot fresh biomass as compared to the un-inoculated control. Whereas the impact of bio inoculants on shoot dry and root fresh weight of maize was also depicted in (**Table 1**). Among sole inoculation, S10 showed a maximum increase in shoot dry biomass followed by ZE27+ZM63+S10 has caused a 27 and 33% increase in shoot dry biomass and root fresh biomass, respectively. Results with respect to the capability of various PGPR strains for improving the root dry biomass of maize are presented in **Table 1**. Maximum root dry weight 7.28 g plant^-1^ was observed under consortia application of PGPR strains. Sole treatments of bacterial strains also exhibited significant improvements in root dry weight. Whereas, among co-inoculation, a significant increase (29%) in root dry biomass was noted under ZM27+S10 co-inoculation, as compared to control.

**Table 1:** Effect of novel bio inoculants on growth parameters of maize in the pot trial.

Treatments	Plant height (cm)	Shoot fresh biomass (g plant^-1^)	Shoot dry biomass (g plant^-1^)	Root fresh biomass (g plant^-1^)	Root dry biomass (g plant^-1^)
Control	73.33 ± 0.12h	37.67 ± 0.19 j	11.72 ± 0.11 e	20.97 ± 0.24 e	5.43 ± 0.21 g
ZM27	74.33 ± 0.14h	42.15 ± 0.23 fg	12.71 ± 0.16 c-e	24.27 ± 0.22 cd	6.00 ± 0.15 ef
ZM31	82.00 ± 0.21d-f	42.90 ± 0.16 e-g	13.02 ± 0.17 c-e	22.83 ± 0.21 d	6.13 ± 0.17 d-f
ZM63	88.00 ± 0.23 b	39.81 ± 0.11 hi	13.56 ± 0.16 a-d	24.10 ± 0.10 d	6.00 ± 0.15 ef
S10	89.33 ± 0.13 b	38.06 ± 0.10 ij	13.93 ± 0.23 a-c	26.93 ± 0.15 ab	5.83 ± 0.21 fg
ZM27+ZM31	84.33 ± 0.14 cd	44.60 ± 0.24 c-e	12.40 ± 0.18 de	24.07 ± 0.13 d	6.37 ± 0.26 c-f
ZM27+ZM63	78.00 ± 0.15 g	41.33 ± 0.21 gh	12.49 ± 0.11 c-e	23.13 ± 0.17 d	6.13 ± 0.23 d-f
ZM27+S10	94.00 ± 0.21 a	47.03 ± 0.19 ab	14.80 ± 0.19 ab	26.50 ± 0.18 ab	7.00 ± 0.21 ab
ZM31+ZM63	88.67 ± 0.18 b	46.44 ± 0.11 a-c	12.31 ± 0.20 de	24.07 ± 0.19 d	5.93 ± 0.18 e-g
ZM31+S10	86.67 ± 0.13 bc	44.50 ± 0.12 c-e	13.37 ± 0.14 b-d	25.73 ± 0.15 bc	6.40 ± 0.26 c-e
ZM63+S10	83.00 ± 0.19 de	43.91 ± 0.13 d-f	12.51 ± 0.12 c-e	23.60 ± 0.16 d	6.25 ± 0.21 d-f
ZM27+ZM31+ZM63	82.00 ± 0.13 d-f	43.80 ± 0.17 d-f	12.92 ± 0.13 c-e	23.30 ± 0.18 d	6.55 ± 0.21 b-d
ZM27+ZM31+S10	79.00 ± 0.21 fg	42.17 ± 0.18 fg	12.31 ± 0.14 de	23.90 ± 0.19 d	6.59 ± 0.16 b-d
ZM27+ZM63+S10	96.00 ± 0.16 a	48.03 ± 0.19 a	14.94 ± 0.15 a	27.80 ± 0.12 a	7.28 ± 0.17 a
ZM31+ZM63+S10	79.67 ± 0.18 e-g	44.58 ± 0.21 c-e	13.06 ± 0.19 c-e	23.17 ± 0.24 d	6.63 ± 0.18 b-d
ZM27+ZM31+ZM63+S10	81.33 ± 0.15 d-g	45.22 ± 0.22 b-d	13.21 ± 0.22 cd	23.26 ± 0.21 d	6.90 ± 0.19 a-c
LSD (*p ≤ 0.05*)	3.5199	2.0915	1.4840	1.6179	0.5420

Means sharing the same letter(s) do not differ significantly at p ≤ 0.05.

#### 3.1.2 Mineral contents of maize

Consortium application of the PGPR strains gave more promising results compared to sole and co-inoculation. An increase in nitrogen content of 4% was found with the sole application of PGPR strains S10. Among co-inoculated treatments, a significant increment in nitrogen contents of 14 and 10% was observed under ZM27+S10 and ZM63+S10, respectively as compared to the un-inoculated control. The maximum increase in nitrogen contents was caused by the application of consortium (ZM27+ZM63+S10) which was 16%, as compared to the un-inoculated treatment. Effects of novel bio inoculant on phosphorous and potassium content in the shoot of maize are also demonstrated in **Table 2**. Treatments with sole application caused a non-significant increment in phosphorous content whereas the treatments with co-inoculation and consortium application have caused significant improvements in shoot phosphorus contents. However, the consortium application (ZM27+ZM63+S10) caused a maximum increment in phosphorous content showing a 23% increase in grain phosphorous. Potassium contents were significantly improved under all application methods, however, the co-inoculation and consortia application indicated more promising outcomes as a maximum increase in potassium contents (18%) was observed under the ZM27+ZM63+S10 consortium application.

**Table 2:** Effect of novel bio inoculants on root dry biomass and macronutrients contents of maize in the pot trial.

Treatments	Nitrogen concentration in shoot (%)	Phosphorous concentration in shoot (%)	Potassium concentration in shoot (%)
Control	2.51 ± 0.16 j	0.48 ± 0.25 e	3.10 ± 0.15 k
ZM27	2.54 ± 0.17 ij	0.49 ± 0.23 e	3.26 ± 0.18 j
ZM31	2.57 ± 0.18 I	0.49 ± 0.25 e	3.38 ± 0.16 h
ZM63	2.55 ± 0.09 I	0.51 ± 0.21 de	3.33 ± 0.15 I
S10	2.62 ± 0.21 h	0.52 ± 0.26 c-e	3.42 ± 0.16 gh
ZM27+ZM31	2.63 ± 0.22 h	0.53 ± 0.28 b-d	3.43 ± 0.16 fg
ZM27+ZM63	2.66 ± 0.23 gh	0.53 ± 0.21 b-d	3.43 ± 0.21 fg
ZM27+S10	2.87 ± 0.25 ab	0.57 ± 0.20 ab	3.58 ± 0.22 b
ZM31+ZM63	2.72 ± 0.24 f	0.55 ± 0.18 b	3.45 ± 0.19 e-g
ZM31+S10	2.68 ± 0.21 fg	0.55 ± 0.18 b	3.48 ± 0.24 d-f
ZM63+S10	2.77 ± 0.22 e	0.54 ± 0.19 b-d	3.49 ± 0.25 de
ZM27+ZM31+ZM63	2.85 ± 0.23 bc	0.55 ± 0.21 bc	3.49 ± 0.14 de
ZM27+ZM31+S10	2.79 ± 0.24 de	0.55 ± 0.16 b	3.52 ± 0.16 cd
ZM27+ZM63+S10	2.90 ± 0.26 a	0.60 ± 0.19 a	3.66 ± 0.19 a
ZM31+ZM63+S10	2.84 ± 0.25 bc	0.54 ± 0.16 b-d	3.54 ± 0.21 bc
ZM27+ZM31+ZM63+S10	2.82 ± 0.27 cd	0.54 ± 0.21 b-d	3.54 ± 0.22 bc
LSD (*p ≤ 0.05*)	0.0415	0.0358	0.0438

Means sharing the same letter(s) do not differ significantly at p ≤ 0.05.

#### 3.1.3 Zn and Fe biofortification

**Figure 1:**
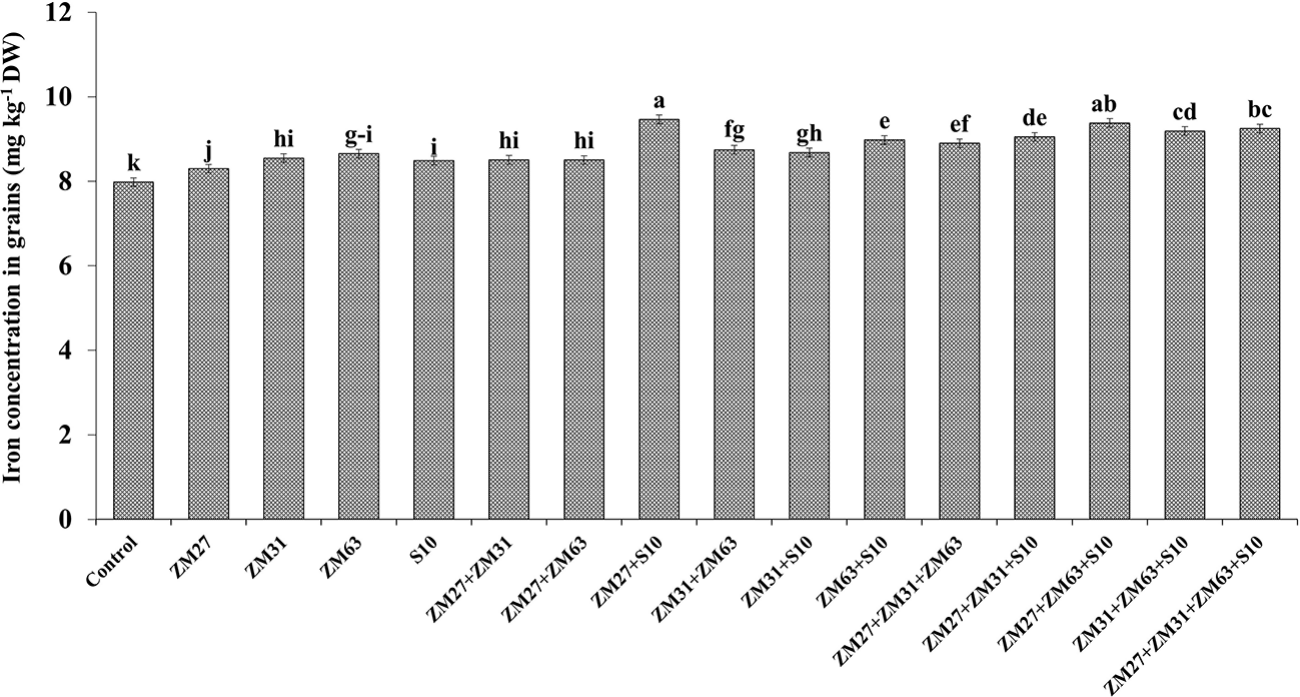
Effect of PGPR inoculation on shoot iron contents in maize in the pot trial. The bars with different letters are significantly different at p ≤ 0.05.

**Figure 2:**
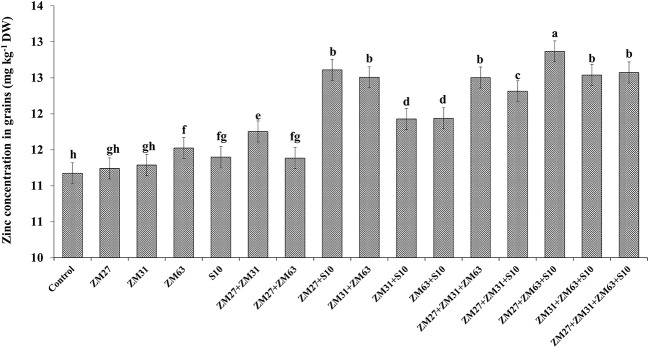
Effect of PGPR inoculation on shoot zinc contents in maize in the pot trial. The bars with different letters are significantly different at p ≤ 0.05.

The efficacy of novel bioinoculants in micronutrient (iron and zinc) uptake in maize shoots is presented in **Figures 1**, **2**, respectively. The application of microbial inoculants significantly improves shoot Zn and Fe in maize as compared to the control treatment. The maximum increase in maize shoot Fe contents (18%) was caused by the consortium inoculation (ZM27+ZM63+S10) as compared to the non-inoculated treatment. Similar results regarding Zn shoot contents were also found under consortium application where ZM27+ZM63+S10 consortium has caused a 15% increase in maize shoot Zn contents followed by co-inoculation of ZM27+S10 where 13% more Zn contents were recorded as compared with the un-inoculated control.

#### 3.1.4 Microbial population

**Figure 3:**
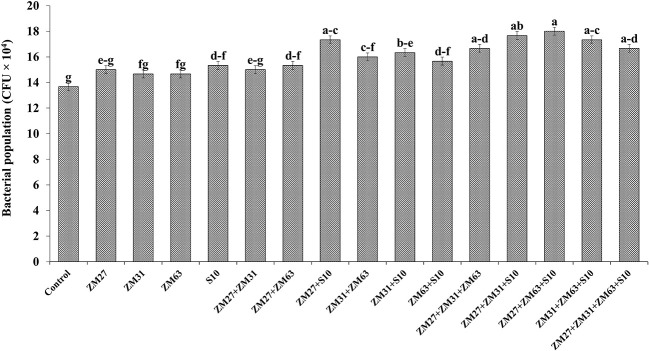
Effect of PGPR inoculation on microbial population in maize rhizosphere in the pot trial. The bars with different letters are significantly different at p ≤ 0.05.

The application methods of novel PGPR strains significantly improved the soil quality in terms of the bacterial population (CFU x 10^4^) in the maize rhizosphere the results are presented in **Figure 3**. Under co-inoculated treatments, ZM27+S10 and ZM63+S10 caused maximum increment (15%) in the bacterial population as compared to the un-inoculated control. The correlation of sole, co, and consortium application revealed that consortia application as ZM27+ZM63+S10 showed the highest increment in microbial population by 23% followed by ZM31+ZM63+S10 that demonstrated a 21% increase in bacterial population as compared to the control treatment.

### 3.2 Field trials

#### 3.2.1 Growth promotion of maize by PGPR application

Different application methods of these promising PGPR strains (sole, co, and consortium application) significantly improved maize growth under both field trials (**Table 3**). Maximum increments in maize shoot lengths of 13 and 16% were caused by the consortium application (ZM27+ZM63+S10) in both field trials, respectively. However, co-inoculation (ZM27+S10) significantly improved the shoot length of maize by 11% in experiment I. Similarly, the PGPR application also significantly enhanced the shoot fresh weight of the maize; the maximum increase in shoot fresh weight was found under the PGPR consortium (ZM27+ZM63+S10) application which was 19 and 23% in field trial I and II, respectively, as compared to the un-inoculated control.

**Table 3:** Effect of novel bio inoculants on plant height and shoot fresh biomass of maize in field trials.

Treatment	Plant height (cm)		Shoot fresh biomass (t ha^-1^)	
	Trial I	Trial II	Trial I	Trial II
Control	218 ± 0.11 h	210 ± 0.20 g	69.3 ± 0.19 g	66.3 ± 0.13 j
ZM27	233 ± 0.18 d-g	215 ± 0.18 fg	71.3 ± 0.14 fg	68.7 ± 0.16 ij
ZM31	231 ± 0.14 fg	217 ± 0.17 ef	71.0 ± 0.17 fg	70.0 ± 0.15 f-I
ZM63	228 ± 0.16 g	215 ± 0.22 fg	75.0 ± 0.`9 de	71.3 ± 0.12 e-h
S10	235 ± 0.21 c-f	216 ± 0.24 e-g	72.7 ± 0.21 e-g	68.3 ± 0.12 ij
ZM27+ZM31	238 ± 0.26 b-e	221 ± 0.19 d-f	71.3 ± 0.16 fg	69.7 ± 0.12 f-I
ZM27+ZM63	237 ± 0.17 b-f	222 ± 0.15 de	74.3 ± 0.18 ef	69.3 ± 0.15 g-I
ZM27+S10	242 ± 0.18 ab	227 ± 0.11 cd	79.3 ± 0.15 a-c	74.3 ± 0.13 b-d
ZM31+ZM63	238 ± 0.11 b-e	230 ± 0.14 c	76.0 ± 0.12 c-e	72.3 ± 0.15 d-f
ZM31+S10	232 ± 0.18 e-g	225 ± 0.15 cd	73.7 ± 0.17 ef	72.0 ± 0.19 d-g
ZM63+S10	239 ± 0.23 b-d	227 ± 0.19 cd	78.0 ± 0.13 b-d	75.7 ± 0.21 a-c
ZM27+ZM31+ZM63	240 ± 0.12 b-d	232 ± 0.14 bc	79.7 ± 0.15 ab	76.0 ± 0.16 a-c
ZM27+ZM31+S10	241 ± 0.19 a-c	230 ± 0.16 bc	78.7 ± 0.11 bc	76.7 ± 0.19 ab
ZM27+ZM63+S10	247 ± 0.21 a	243 ± 0.13 a	82.7 ± 0.18 a	77.7 ± 0.12 a
ZM31+ZM63+S10	244 ± 0.22 ab	237 ± 0.16 ab	81.0 ± 0.19 ab	73.3 ± 0.15 c-e
ZM27+ZM31+ZM63+S10	244 ± 0.28 ab	241 ± 0.18 a	79.0 ± 0.15 bc	73.3 ± 0.19 c-e
LSD (*p ≤ 0.05*)	6.9903	6.9768	3.5591	2.9168

Means sharing the same letter(s) do not differ significantly at p ≤ 0.05.

The PGPR inoculants improved the shoot dry biomass of maize under field conditions (**Table 4**). The data depicted that the sole inoculation of PGPR has improved by 13% shoot dry weight, while co-inoculation improved by 21% as compared to control. However, the maximum increase in shoot dry weight was observed under the consortium application of PGPR which was found 26% more than the un-inoculated control.

**Table 4:** Effect of novel bio inoculants on shoot dry biomass and SPAD value of maize in field trials.

Treatment	Shoot dry biomass (t ha^-1^)		SPAD chlorophyll value	
	Trial I	Trial II	Trial I	Trial II
Control	12.9 ± 0.14 d	11.2 ± 0.16 e	35.3 ± 0.16 f	34.7 ± 0.18 g
ZM27	13.0 ± 0.15 d	12.4 ± 0.07 a-e	38.0 ± 0.16 b-f	36.7 ± 0.19 e-g
ZM31	13.2 ± 0.17 cd	11.5 ± 0.23 de	35.7 ± 0.21 ef	36.0 ± 0.21 fg
ZM63	13.0 ± 0.13 cd	11.8 ± 0.13 c-	37.3 ± 0. 23c-f	36.7 ± 0.13 e-g
S10	14.6 ± 0.15 a-d	12.5 ± 0.14 a-e	37.0 ± 0.14 d-f	38.7 ± 0.15 c-e
ZM27+ZM31	14.6 ± 0.18 a-d	12.0 ± 0.15 b-e	39.0 ± 0.16 a-f	37.7 ± 0.15 d-f
ZM27+ZM63	13.5 ± 0.12 b-d	12.0 ± 0.11 b-e	38.7 ± 0.15 a-f	37.3 ± 0.13 d-f
ZM27+S10	15.3 ± 0.21 a-c	13.4 ± 0.09 ab	41.7 ± 0.11 a-c	40.3 ± 0.17 a-c
ZM31+ZM63	13.3 ± 0.19 cd	12.3 ± 0.15 b-e	39.7 ± 0.27 a-f	38.7 ± 0.15 c-e
ZM31+S10	15.7 ± 0.17 a	13.2 ± 0.09 a-c	40.7 ± 0.13 a-d	39.3 ± 0.15 b-d
ZM63+S10	15.5 ± 0.16 ab	12.7 ± 0.18 a-d	39.0 ± 0.13 a-f	39.0 ± 0.16 b-ds
ZM27+ZM31+ZM63	15.5 ± 0.13 a-c	13.1 ± 0.21 a-c	40.0 ± 0.16 a-e	41.0 ± 0.17 ab
ZM27+ZM31+S10	15.3 ± 0.14 a-c	13.2 ± 0.11 a-c	40.7 ± 0.14 a-d	40.3 ± 0.17 a-c
ZM27+ZM63+S10	16.3 ± 0.19 a	13.8 ± 0.14 a	42.7 ± 0.11 a	42.0 ± 0.14 a
ZM31+ZM63+S10	15.2 ± 0.22 a-c	13.2 ± 0.14 a-c	42.3 ± 0.10 ab	41.0 ± 0.25 ab
ZM27+ZM31+ZM63+S10	14.4 ± 0.24 a-d	12.8 ± 0.15 a-d	40.7 ± 0.15 a-d	40.0 ± 0.15 a-c
LSD (*p ≤ 0.05*)	2.1668	1.4484	4.4593	2.2085

Means sharing the same letter(s) do not differ significantly at p ≤ 0.05.

#### 3.2.2 Effect of PGPR on physiology and yield of maize

Results regarding the SPAD value of maize showed a significant increment under all application methods of novel PGPR strains (**Table 4**). The maximum increase in SPAD value under sole application was caused by S10 which was 12% higher than the control treatment in field trial II. However, the co-inoculation of ZM27+S10 showed a maximum increase in SPAD contents by 18% in field trial I which was at par with the consortium application of ZM27+ZM31+ZM63 in trial II. The consortium application caused the maximum increase in SPAD value under the M27+ZM63+S10 application which was 21% more than the un-inoculated control in both field trials.

Sole, co, and consortium PGPR strains caused significant improvement in the maize grain quality in terms of the number of grains cob^-1^ and 1000 grain weight (**Table 5**). The results indicated that sole inoculation of bacterial strains ZM27 in field trial I and S10 in field trial II caused a 2% increase in the number of grains cob^-1^ as compared to the control. Whereas S10 showed a 4% increase in 1000 grain weight followed by ZM63 with a 3% increase as compared to the control. The co-inoculation and consortia application indicated significantly higher results than sole inoculation. Co-inoculation of ZM27+S10 in both field trials and consortia treatment of ZM27+ZM63+S10 in trial II increased the number of grains cob^-1^ by 4 and 6%, respectively. Besides the individual use of PGPR, co-inoculation of ZM27+S10 showed 8% and consortia application of compatible bacterial strains (ZM27+ZM63+S10) caused a 12% increase in 1000 grain weight of maize. The results regarding grain yield depicted that the use of PGPR strains improved the grain yield of maize crop under field conditions (**Table 6**). A maximum increment in grain yield was observed in the consortium (ZM27+ZM63+S10) applied treatment, which was 17% more as compared to the un-inoculated control. However, co-inoculation of ZM27+S10 showed a 13% increase in grain yield, as compared to control in trial II.

**Table 5:** Effect of novel bio inoculants on grain number and weight of maize in field trials.

Treatment	Number of grains cob^-1^		1000 grain weight (g)	
	Trial I	Trial II	Trial I	Trial II
Control	475 ± 0.12 i	468 ± 0.15 j	258 ± 0.15 h	250 ± 0.15 h
ZM27	482 ± 0.14 g	471 ± 0.12 I	260 ± 0.14 gh	255 ± 0.15 gh
ZM31	480 ± 0.15 h	473 ± 0.12 h	264 ± 0.14 fg	256 ± 0.19 fg
ZM63	480 ± 0.14 gh	475 ± 0.15 h	266 ± 0.13 f	258 ± 0.18 e-g
S10	481 ± 0.13 gh	477 ± 0.15 g	264 ± 0.19 fg	260 ± 0.16 ef
ZM27+ZM31	482 ± 0.15 gh	479 ± 0.16 fg	265 ± 0.13 f	257 ± 0.18 e-g
ZM27+ZM63	482 ± 0.16 g	481 ± 0.17 ef	267 ± 0.19 f	261 ± 0.16 de
ZM27+S10	491 ± 0.16 cd	485 ± 0.12 c	278 ± 0.13 b-d	271 ± 0.19 b
ZM31+ZM63	487 ± 0.16 f	481 ± 0.21 de	273 ± 0.09 e	266 ± 0.21 cd
ZM31+S10	488 ± 0.12 ef	479 ± 0.25 ef	274 ± 0.12 de	269 ± 0.12 bc
ZM63+S10	490 ± 0.14 de	486 ± 0.19 c	274 ± 0.14 de	268 ± 0.15 bc
ZM27+ZM31+ZM63	494 ± 0.16 b	483 ± 0.21 d	279 ± 0.12 b-d	270 ± 0.11 bc
ZM27+ZM31+S10	492 ± 0.18 bc	480 ± 0.22 ef	280 ± 0.15 bc	269 ± 0.12 bc
ZM27+ZM63+S10	499 ± 0.19 a	495 ± 0.14 a	290 ± 0.17 a	276 ± 0.18 a
ZM31+ZM63+S10	491 ± 0.21 cd	489 ± 0.11 b	283 ± 0.12 b	272 ± 0.19 ab
ZM27+ZM31+ZM63+S10	489 ± 0.13 d-f	485 ± 0.10 c	277 ± 0.14 c-e	267 ± 0.14 bc
LSD (*p ≤ 0.05*)	2.1821	2.1874	5.0597	4.8265

Means sharing the same letter(s) do not differ significantly at p ≤ 0.05.

**Table 6:** Effect of novel bio inoculants on Grain yield and N contents of Maize in field trial.

Treatment	Grain yield (t ha^-1^)		Nitrogen concentration in grains (%)	
	Trial I	Trial II	Trial I	Trial II
Control	8.60 ± 0.12 d	8.72 ± 0.12 h	2.48 ± 0.12 i	2.42 ± 0.21 h
ZM27	8.93 ± 0.14 cd	9.09 ± 0.15 f-h	2.52 ± 0.11 hi	2.46 ± 0.25 f-h
ZM31	8.89 ± 0.15 cd	8.98 ± 0.13 gh	2.54 ± 0.10 gh	2.44 ± 0.23 gh
ZM63	9.07 ± 0.18 b-d	9.19 ± 0.21 e-g	2.53 ± 0.19 h	2.48 ± 0.18 e-g
S10	9.15 ± 0.19 b-d	9.01 ± 0.14 f-h	2.57 ± 0.20 fg	2.49 ± 0.19 ef
ZM27+ZM31	9.28 ± 0.13 a-d	9.39 ± 0.22 d-g	2.60 ± 0.21 ef	2.51 ± 0.13 de
ZM27+ZM63	9.46 ± 0.17 a-c	9.43 ± 0.19 c-f	2.64 ± 0.17 e	2.54 ± 0.18 d
ZM27+S10	9.65 ± 0.18 a-c	9.83 ± 0.13 a-c	2.72 ± 0.19 cd	2.63 ± 0.21 bc
ZM31+ZM63	9.42 ± 0.19 a-d	9.79 ± 0.18 a-d	2.69 ± 0.13 d	2.63 ± 0.14 bc
ZM31+S10	9.64 ± 0.21 a-c	9.71 ± 0.19 a-d	2.70 ± 0.12 d	2.61 ± 0.26 c
ZM63+S10	9.47 ± 0.22 a-c	9.90 ± 0.16 ab	2.73 ± 0.18 cd	2.61 ± 0.12 c
ZM27+ZM31+ZM63	9.60 ± 0.21 a-c	9.59 ± 0.13 b-e	2.75 ± 0.17 bc	2.59 ± 0.17 c
ZM27+ZM31+S10	9.76 ± 0.16 ab	9.77 ± 0.12 a-d	2.75 ± 0.15 bc	2.60 ± 0.16 c
ZM27+ZM63+S10	10.07 ± 0.17 a	10.09 ± 0.15 a	2.80 ± 0.13 a	2.69 ± 0.23 a
ZM31+ZM63+S10	9.81 ± 0.18 ab	9.87 ± 0.14 ab	2.78 ± 0.15 ab	2.66 ± 0.24 ab
ZM27+ZM31+ZM63+S10	9.61 ± 0.19 a-c	9.83 ± 0.18 a-c	2.74 ± 0.18 c	2.61 ± 0.21 c
LSD (*p ≤ 0.05*)	0.8234	0.4249	0.0398	0.0449

Means sharing the same letter(s) do not differ significantly at p ≤ 0.05.

#### 3.2.3 Effect of PGPR inoculation on mineral contents of maize

Results about the impact of PGPR strains by different application methods on nitrogen content in grains are presented in **Table 7** which showed a significant increase as compared to the un-inoculated control. The consortium of ZM27+ZM63+S10 strains has caused the highest increase of 13% in nitrogen content in grains of maize under both field conditions. However, the consortium application also has caused the highest increase in the case of phosphorus and potassium contents (**Table 7**). The highest increase in phosphorus and nitrogen content of maize grain was found at 12 and 10%, respectively, under consortium (ZM27+ZM63+S10) treatment as compared to the un-inoculated control treatment.

**Table 7:** Effect of novel bio inoculants on phosphorous and potassium concentration in the shoot of maize in field trials.

Treatment	Phosphorous concentration in grains (%)		Potassium concentration in grains (%)	
	Trial I	Trial II	Trial I	Trial II
Control	0.45 ± 0.24 g	0.42 ± 0.16 e	2.13 ± 0.19 h	2.46 ± 0.16 i
ZM27	0.46 ± 0.26 e-g	0.42 ± 0.17 de	2.17 ± 0.22 g	2.51 ± 0.14 h
ZM31	0.47 ± 0.27 d-g	0.42 ± 0.19 c-e	2.17 ± 0.14 gh	2.53 ± 0.12 gh
ZM63	0.47 ± 0.23 b-g	0.43 ± 0.14 a-e	2.22 ± 0.14 ef	2.49 ± 0.13 hi
S10	0.46 ± 0.21 fg	0.42 ± 0.16 c-e	2.23 ± 0.18 ef	2.52 ± 0.17 gh
ZM27+ZM31	0.47 ± 0.13 c-g	0.43 ± 0.13 b-e	2.19 ± 0.14 fg	2.55 ± 0.12 fg
ZM27+ZM63	0.48 ± 0.11 b-f	0.44 ± 0.13 a-e	2.22 ± 0.15 ef	2.57 ± 0.15 ef
ZM27+S10	0.48 ± 0.10 a-e	0.45 ± 0.18 a-e	2.27 ± 0.13 cd	2.62 ± 0.21 cd
ZM31+ZM63	0.48 ± 0.09 b-f	0.44 ± 0.16 a-e	2.24 ± 0.19 de	2.60 ± 0.19 de
ZM31+S10	0.48 ± 0.11 a-f	0.43 ± 0.19 a-e	2.25 ± 0.14 de	2.62 ± 0.14 cd
ZM63+S10	0.48 ± 0.21 a-e	0.45 ± 0.16 a-e	2.28 ± 0.12 cd	2.65 ± 0.11 bc
ZM27+ZM31+ZM63	0.48 ± 0.06 b-f	0.45 ± 0.12 a-c	2.28 ± 0.21 cd	2.63 ± 0.10 cd
ZM27+ZM31+S10	0.50 ± 0.13 ab	0.45 ± 0.15 a-d	2.31 ± 0.14 bc	2.68 ± 0.06 ab
ZM27+ZM63+S10	0.50 ± 0.14 a	0.46 ± 0.15 a	2.35 ± 0.21 a	2.72 ± 0.15 a
ZM31+ZM63+S10	0.49 ± 0.17 a-c	0.46 ± 0.19 ab	2.33 ± 0.14 ab	2.69 ± 0.12 a
ZM27+ZM31+ZM63+S10	0.49 ± 0.19 a-d	0.45 ± 0.18 a-e	2.29 ± 0.17 bc	2.64 ± 0.15 cd
LSD (*p ≤ 0.05*)	0.0261	0.0301	0.0384	0.0419

Means sharing the same letter(s) do not differ significantly at p ≤ 0.05.

**Figure 4:**
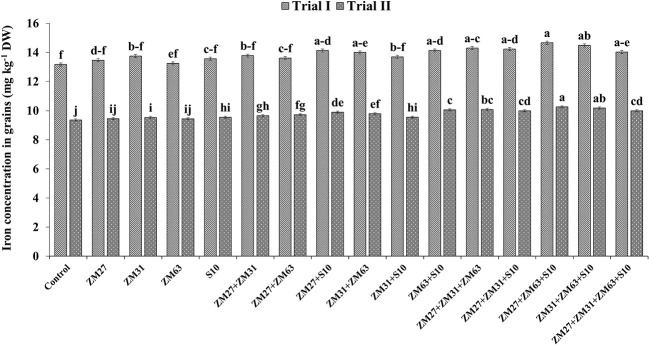
Effect of PGPR inoculation on grain iron contents in maize in the pot trial. The bars with different letters are significantly different at p ≤ 0.05.

**Figure 5:**
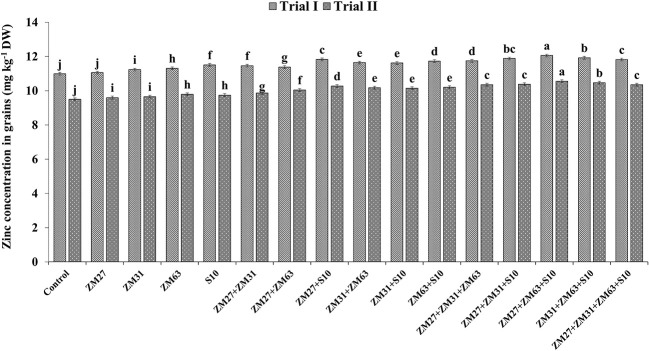
Effect of PGPR inoculation on grain zinc contents in maize in the pot trial. The bars with different letters are significantly different at p ≤ 0.05.

Results regarding the effect of different PGPR inoculation methods on micronutrient biofortification in maize grain depicted a significant increase in the uptake and biofortification of Fe and Zn in maize grain presented in **Figures 4**, **5**, respectively. The co-inoculation of ZM27+S10 and ZM63+S10 showed a significant increase of 7% in Fe contents as compared to the control treatment. Moreover, the consortium application (ZM27+ZM63+S10) has caused 11. 4% more Fe concentration in maize grain under field trial. Similarly, the consortium of bacterial strains (ZM27+ZM63+S10) has caused significant improvement in maize grain zinc content by 11.6 and 10.0% in field trials II and I, respectively, as compared to the un-inoculated control treatment.

#### 3.2.4 Effect of PGPR inoculation on rhizosphere microbial population of maize

**Figure 6:**
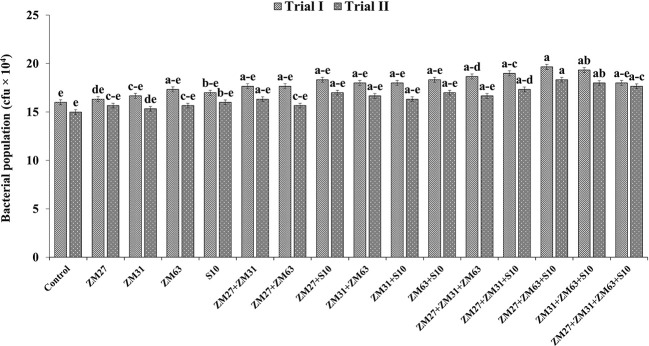
Effect of PGPR inoculation on the microbial count in maize rhizosphere in the field trials. The bars with different letters are significantly different at p ≤ 0.05.

The application of Zn solubilizing PGPR by different methods (sole, co, and consortium application) significantly increased the microbial population in the maize rhizosphere as compared to the un-inoculated control (**Figure 6**). The sole inoculation has caused the lowest increase (6.2 and 8.1%) in the microbial population in maize rhizosphere by ZM63 and S10, respectively, as compared to the uninoculated control in filed trial I. The co-inoculation of ZM27+S10 has caused the maximum increase in microbial population (15%) with respect to the control treatment. The maximum increment (23%) in the bacterial population under consortium application was caused by ZM27+ZM63+S10 in field trial I followed by ZM31+ZM63+S10 which showed a 21% increase in bacterial population over the control.

## 4 Discussion

The current investigation revealed the role of Zn solubilizing PGPR and their sole, co, and consortium application on maize growth, yield, and micronutrients (Fe and Zn) biofortification. The results of the present study showed that all methods of biofertilizer application (sole, co, and consortium) increase the growth, yield, and nutrient status of the maize but the most promising results were obtained from the consortium (ZM31+ZM63+S10) application. The microbial inoculation of maize with PGPR strains i.e. *Bacillus subtilis*. ZM63, *Bacillus aryabhattai*. ZM31, *Bacillus aryabhattai*. S10 and *Paenibacillus polymyxa*. ZM27 has increased plant development, just as improving mineral nutrients in shoots and grains of maize. A positive relationship between plant biomass and nutrient content (N, P, Fe) was also recorded.

The role of microbial inoculants in enhancing maize growth and biofortification has already been described the microbial community does so through the degradation of organic matter and releasing entrapped nutrients which add up to the soil fertility status, and in turn, is responsible for improved plant growth (McDonagh et al., 1993; Phoomthaisong et al., 2003; Zou et al., 2019). The microorganisms solubilize the mineral nutrients by releasing organic acids into the rhizosphere and reducing the microsite pH suitable for micronutrients availability (Fe and Zn) and bio-fortify maize (Hussain et al., 2019; Merinero et al., 2022). Moreover, the increase in plant height, and shoot fresh and dry biomass of maize with inoculation of PGPR in the present study were at par with Khalid et al. (2004) and Pereira et al. (2020) and they conclude that the improvement was due to the growth hormones related substance produced by PGPR and better nutrient status of maize. The improvement in plant height, and shoot and root dry biomasses might be due to the enhanced nutrient uptake and partitioning especially higher nitrogen and phosphorus are responsible for growth and development at the early stage of the maize (Aquino et al., 2021). Pearson’s correlation of the growth parameters and nutrient contents of maize in pot trial and field is presented in supplementary data (**Tables S2-S4**) and also revealed that there was a positive correlation between the mineral nutrients uptake and maize growth parameters, which justifies the increased growth of maize attributed to the more mineral uptake.

Our previous investigations Ahmad et al. (2014); Mumtaz et al. (2017); Hussain et al. (2020), and Zahir et al. (2011) also advocated the role of the PGPR in enhancing the growth of different crops by enhancing the uptake of the entrapped nutrients through the release of organic acids. Moreover, various other studies also described the role of biofertilizers in improving the vegetative growth of different crops by enhancing nutrient uptake of crops and their partitioning among the different plant parts (Samavat et al., 2012), and biofortify micronutrients to cope with food insecurity and malnutrition (Ahmad et al., 2008; Farooq et al., 2009; Zafar-ul-Hye et al., 2013). Other growth-promoting traits such as auxin (IAA) production by these microbes is responsible for better root infrastructure development and proliferation in the soil, which is a possible reason for root biomass production in the present experiment (Zeb et al., 2018; Ahmad et al., 2019).

The increase in micronutrients (Fe and Zn) was significant under consortium application as compared to the sole and co-inoculation of the PGPR. The result of the present study is in line with Khan et al. (2019) for Zn biofortification while at par with Mumtaz et al. (2022) for Fe biofortification in maize grains. The higher uptake of the described micronutrients was due to the solubilization of these fixed micronutrients by the application of microorganisms, which are responsible for excreting acidic substances (organic acids) in the maize rhizosphere and lowering the microsite pH of soil. The lowering of pH is responsible for the solubilization of these micronutrients and their uptake in the maize plants and their fortification in maize grain (Sheikh et al., 2022). On the other hand, the microorganisms produce specified compounds like siderophores (Fe-loving compounds) which quench the Fe and the other micronutrients and made them available for plant use and reduce their uptake by pathogens (Singh and Prasanna, 2020). Therefore, the biofortification of Fe and Zn in the present investigation might be due to the production of siderophores by these bacterial strains.

The improvement in vegetative growth is responsible for enhancing crop production and higher outcomes from the same piece of land. The result regarding the grains per cob is slightly higher than Chen et al. (2022) which was due to the better growth of the maize due to optimal nutrition and biofertilizer solubilization of the fixed nutrients in the soil and their translocation to the reproductive organs of maize which is responsible for the increase in size and number of grains of the cobs (Amanullah et al., 2021; Inyang et al., 2021). The 1000-grain weight of the maize in the present experiment was at par with the Magar et al. (2021) which depicted that the grain weight improvement is linked with the optimal nutrition of the maize crop and its partitioning among the grains which results in an increased number of grains per cob and increased grain weight. A similar reason for the higher number of grains and thousand-grain weight has also been reported by many researchers (Naveed et al., 2008; Drury et al., 2017; Anwar et al., 2021; Drulis et al., 2022). The results regarding the yield of the present investigation are in line with the Mumtaz et al. (2022) which were due to the higher nutrient uptake, partitioning, and reproductive growth of maize (Al-Suhaibani et al., 2021; Singh et al., 2021; Ebrahimi Chamani et al., 2022). Pearson’s correlation analysis data between growth, nutrient contents, and yield parameters of maize under Field trials are presented in the supplementary data section as **Tables S3**, **S4**, which depicted a strong positive correlation between nutrients concentration, growth, and yield improvements in maize by application of these novel strains application under different methods.

## 5 Conclusions

It can be concluded that the sole, co, and consortium application of the PGPR significantly increases the growth, development, nutritional status, and yield of maize. The PGPR strains are responsible for solubilizing the essential nutrients in maize nitrogen, phosphorous, iron, zinc, and potassium while we compared them to the non-inoculated set of treatments. The results depicted that the application of the PGPR consortium (ZM27+ZM63+S10) the results was synergistic and caused a significant increase in shoot and root biomasses, nutrient status, and yield of maize when compared to un-inoculated control treatment. So, it is not wrong to say that the best consortium in the present study has the potential to be commercialized as a biofertilizer for biofortification (Fe and Zn) in wheat and sustainable production of maize.

## Data availability statement

The original contributions presented in the study are included in the article/**Supplementary Material**. Further inquiries can be directed to the corresponding authors.

## Author contributions

Conceptualization, MA and AH. Methodology, MA, ADa, and ML. Software, AH. Validation, AH, ADi, ADa and ML. Formal analysis, MA, ADi, ADa, ML, and ZI. Funding, AE-S. Investigation, MA, AH, ADa, ML, ZI, HA and FN. Resources, AH, ADa, ADi, AE-S, and ML. Data curation, ML and ZI. Writing—original draft preparation, MA. Writing—review and editing, ADa, ADi, AE-S, and ML. Visualization, AH, ZI, ADi, AE-S, and FN. Supervision, MA and AZ. All authors contributed to the article and approved the submitted version.
