# Supplementary material for: Combating iron and zinc malnutrition through mineral biofortification in maize through plant growth promoting Bacillus and Paenibacillus species
Source: Front Plant Sci. 2023 Feb 1;13:1094551. doi: 10.3389/fpls.2022.1094551 (PMC9929565; doi:10.3389/fpls.2022.1094551)
Supplement: Supplementary file 1 [file Table_1.docx]

**Supplementary Table S1: Physico-chemical characteristics of the soil**

| **Parameter** | **Unit** | **Pot trials** | **Field trial I** | **Field trial II** |
| --- | --- | --- | --- | --- |
| **EC_e_** | dS m^-1^ | 1.9 | 2.1 | 1.9 |
| **pH** | --- | 7.8 | 8.1 | 7.9 |
| **Organic matter** | % | 0.43 | 0.41 | 0.39 |
| **N** | % | 0.058 | 0.048 | 0.043 |
| **P** | mg kg^-1^ | 4.0 | 3.7 | 4.2 |
| **K** | mg kg^-1^ | 81 | 63 | 71 |
| **Zn (DTPA)** | mg kg^-1^ | 0.85 | 0.81 | 0.79 |
| **Fe (DTPA)** | mg kg^-1^ | 4.1 | 3.8 | 4.3 |
| **Saturation percentage** | % | 35 | 35 | 36 |
| **Textural class** | --- | Sandy loam | Sandy loam | Sandy loam |

**Supplementary Table S2: Pearson’s correlation between growth and nutrient contents of maize in pot trial**

| **Parameters** | **Zn contents** | **Shoot Fresh weight** | **Shoot Dry weight** | **Root Dry weight** | **Plant height** | **P-contents** | **N-contents** | **K contents** |
| --- | --- | --- | --- | --- | --- | --- | --- | --- |
| **Shoot Fresh weight** | 0.8048 |  |  |  |  |  |  |  |
| **Shoot dry weight** | 0.4205 | 0.4051 |  |  |  |  |  |  |
| **Root Dry weight** | 0.3610 | 0.3898 | 0.8376 |  |  |  |  |  |
| **Plant Height** | 0.5026 | 0.5135 | 0.8084 | 0.8272 |  |  |  |  |
| **P-contents** | 0.8761 | 0.7665 | 0.5664 | 0.6346 | 0.6870 |  |  |  |
| **N- contents** | 0.9378 | 0.7393 | 0.4516 | 0.3699 | 0.4360 | 0.8859 |  |  |
| **K- contents** | 0.8329 | 0.7690 | 0.5890 | 0.5843 | 0.6209 | 0.9050 | 0.8944 |  |
| **Fe-contents** | 0.8920 | 0.7504 | 0.6078 | 0.4536 | 0.5543 | 0.8315 | 0.9239 | 0.9114 |

**Supplementary Table S3: Pearson’s correlation between growth, nutrient contents and yield parameters of maize under Field trial I**

| **Parameters** | **Zn contents** | **Plant Height** | **P-contents** | **N-contents** | **K contents** | **Fe- contents** | **Shoot fresh biomass** | **Shoot dry biomass** | **Grain yield** |
| --- | --- | --- | --- | --- | --- | --- | --- | --- | --- |
| **Plant Height** | 0.8733 |  |  |  |  |  |  |  |  |
| **P-contents** | 0.8862 | 0.8233 |  |  |  |  |  |  |  |
| **N- contents** | 0.9601 | 0.8556 | 0.9039 |  |  |  |  |  |  |
| **K- contents** | 0.9650 | 0.8446 | 0.8996 | 0.9398 |  |  |  |  |  |
| **Fe-contents** | 0.9084 | 0.8790 | 0.8425 | 0.9211 | 0.8801 |  |  |  |  |
| **Shoot fresh biomass** | 0.9187 | 0.8229 | 0.8579 | 0.9117 | 0.9552 | 0.8793 |  |  |  |
| **Shoot dry biomass** | 0.8458 | 0.6815 | 0.6624 | 0.8231 | 0.8046 | 0.7805 | 0.7118 |  |  |
| **Grain Yield** | 0.9505 | 0.8738 | 0.9229 | 0.9588 | 0.9422 | 0.8688 | 0.8859 | 0.8272 |  |
| **Chlorophyll (SPAD)** | 08932 | 0.8432 | 0.8441 | 0.9128 | 0.8785 | 0.8325 | 0.8559 | 0.7669 | 0.9492 |

**Supplementary Table S4: Pearson’s correlation between growth, nutrient contents and yield parameters of maize under Field trial II**

| **Parameters** | **Zn contents** | **Plant Height** | **P-contents** | **N-contents** | **K contents** | **Fe- contents** | **Shoot fresh biomass** | **Shoot dry biomass** | **Grain yield** |
| --- | --- | --- | --- | --- | --- | --- | --- | --- | --- |
| **Plant Height** | 0.9475 |  |  |  |  |  |  |  |  |
| **P-contents** | 0.9523 | 0.9003 |  |  |  |  |  |  |  |
| **N- contents** | 0.9616 | 0.9098 | 0.8993 |  |  |  |  |  |  |
| **K- contents** | 0.9622 | 0.9313 | 0.9108 | 0.9370 |  |  |  |  |  |
| **Fe-contents** | 0.9281 | 0.9179 | 0.9583 | 0.8681 | 0.9319 |  |  |  |  |
| **Shoot fresh biomass** | 0.8889 | 0.8172 | 0.8647 | 0.8297 | 0.8882 | 0.8699 |  |  |  |
| **Shoot dry biomass** | 0.8492 | 0.7806 | 0.7537 | 0.8567 | 0.8633 | 0.7682 | 0.8000 |  |  |
| **Grain Yield** | 0.9479 | 0.8990 | 0.9004 | 0.9746 | 0.9362 | 0.8647 | 0.8637 | 0.8192 |  |
| **Chlorophyll (SPAD)** | 0.9378 | 0.8907 | 0.8538 | 0.9098 | 0.9151 | 0.8842 | 0.8540 | 0.9473 | 0.8681 |
